# Metagenomic data on the composition of bacterial communities in lake environment sediments for fish farming by next generation Illumina sequencing

**DOI:** 10.1016/j.dib.2020.106228

**Published:** 2020-08-24

**Authors:** María Custodio, Alberto Ordinola-Zapata, Ciro Espinoza, Enedia Vieyra-Peña, Richard Peñaloza, Héctor Sánchez-Suárez, Tessy Peralta-Ortiz

**Affiliations:** a Facultad de Medicina Humana, Centro de Investigación de Medicina en Altura y Medio Ambiente, Universidad Nacional del Centro del Perú, Av. Mariscal Castilla N° 3909, Huancayo, Perú; b Facultad de Ingeniería Pesquera y Ciencias del Mar, Universidad Nacional de Tumbes, Calle Los Ceibos S/N, Puerto Pizarro, Tumbes, Perú; c Facultad de Ciencias Agrarias, Departamento Académico de Medicina Veterinaria y Zootecnia, Universidad Nacional de Tumbes, La Cruz S/N, Tumbes, Perú

**Keywords:** Gen 16S rRNA, Bacterial composition, Sediment, Gaps, Fish farming

## Abstract

This article contains data on the bacterial communities of lagoon sediments with fish potential in the Central Andes of Peru. The surface sediment samples were collected from four lagoons destined for continental water fish farming. DNA extraction was performed from 0.5 g of sample through the Presto™ Soil DNA Extraction Kit. Bacterial sequencing of the 16S rRNA amplicon was performed on the DNA extracted from the sediment. At least 36 Phyla bacteria were detected, the bacterial communities being dominated by Proteobacteria, Cyanobacteria, Actinobacteria, Firmicutes, Chloroflexi. These data can be used for predictive analysis to gain a better understanding of the dynamics of bacterial communities in environments under pressure from fish farming.

## Specifications Table

**Table:** 

Subject	Biology
Specific subject area	Microbial ecology
Type of data	Tables, figures, FASTQ
How data was acquired	High performance sequencing data of the 16S rRNA gene amplicon using Illumina MiSeq sequencing [1].
Data format	Raw and analyzed
Parameters for data collection	Identification of ponds with fish activity and sediment collection.
Description of data collection	Extraction and amplification of bacterial DNA by PCR and sequencing of 16S bacterial rRNA amplicon [2].
Data source location	Lagoons with fish potential located in the Central Andes of Peru, between latitude −11.7808°, longitude −75.2454° and latitude −11.7198, longitude −75.2311 (Fig. 1).
Data accessibility	Data is available in the article.

## Value of the Data

• These data are the first generated using 16S rRNA genes from bacterial communities in lake environments pressured by fish farming in the Peruvian Andes.
• These metagenomic data may be useful to other researchers to expand molecular studies and compare the composition of bacterial communities under different environmental and anthropogenic factors.
• These data can be used for predictive analysis to gain a better understanding of the dynamics of bacterial communities in environments under pressure from fish farming.

## 1 Data Description

### 1.1 Study area

**Fig. 1:**
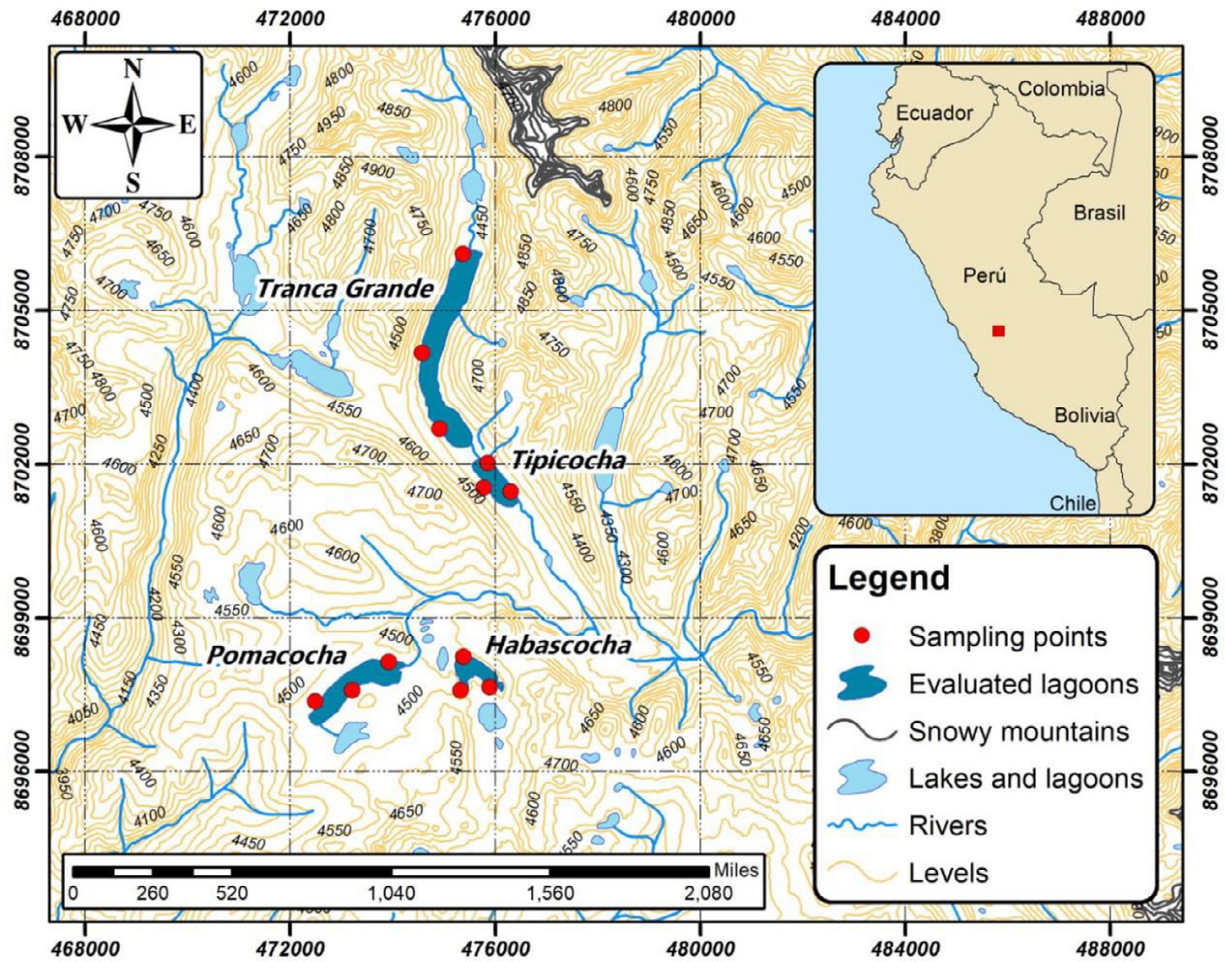
Location map of the study area in the Perene river watershed, Peru.

The study was conducted in the Pomacocha, Habascocha, Tipicocha and Tranca Grande lagoons of glacial origin located in the Central Andes of Peru, in the upper basin of the Perene River, at an altitude between 4310 and 4330 m.a.s.l. [3]. The four lagoons are used for intensive farming of *Oncorhynchus mykiss* (rainbow trout) in large floating cages (Fig. 1).

### 1.2 Analytical data

**Fig. 2:**
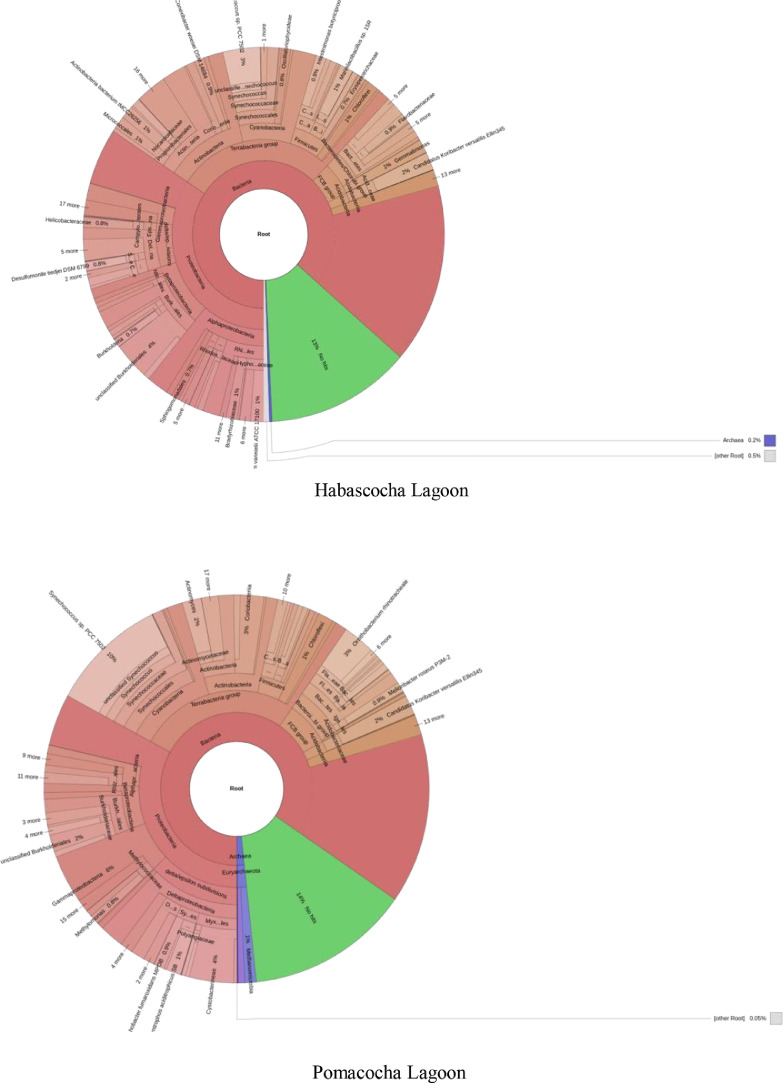

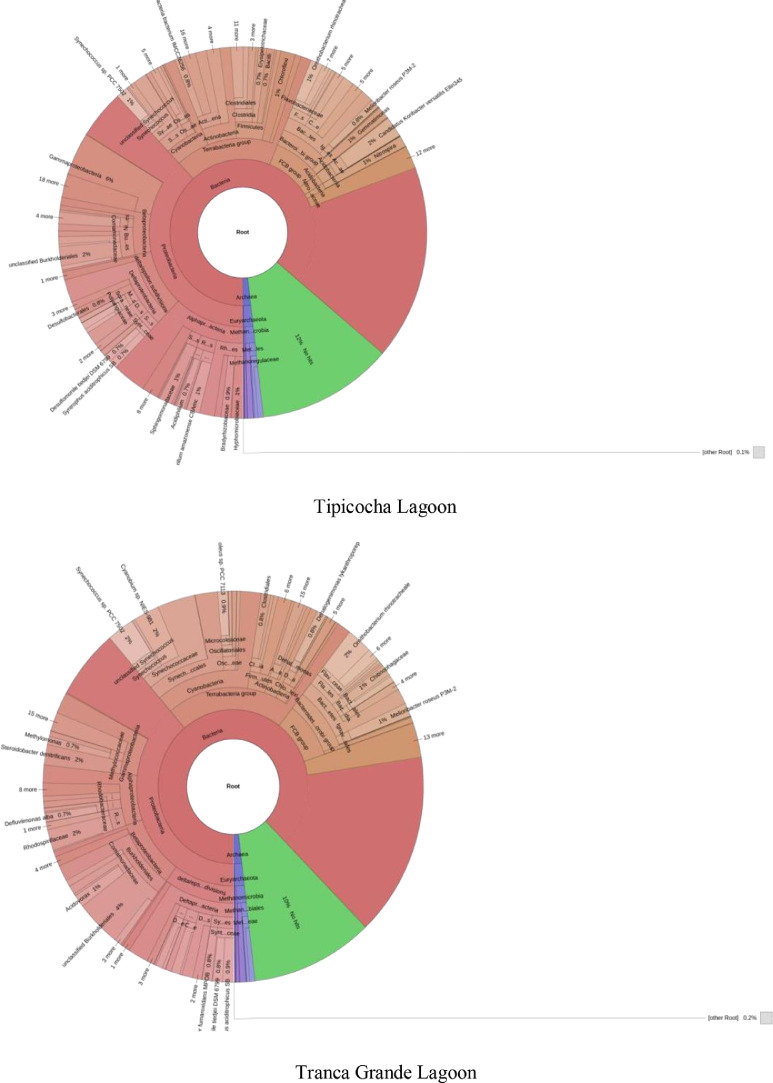
Composition of bacterial communities in lake sediments with fish potential in the Central Andes of Peru.

The metagenomic data presented in this manuscript provide information on the bacterial communities of lagoon sediments intended for the cultivation of *Oncorhynchus mykiss* in the Central Andes of Peru. The bacterial taxonomic composition generated through sequencing of the 16S rRNA amplicon using the standard next-generation Illumina MiSeq protocol is shown in Fig. 2. Analysis of the final readings revealed the Bacteria and Archaea domains. In the Habascocha lagoon the readings revealed 33 phyla, 64 classes and 127 orders, in the Pomacocha lagoon 30 phyla, 61 classes and 120 orders, in the Tipicocha lagoon 34 phyla, 61 classes and 130 orders and, in the Tranca Grande lagoon 31 phyla, 55 classes and 127 orders. The readings also revealed 276 bacterial families in the four lakes. However, between 10% and 14% of the total readings were not classified.

**Fig. 3:**
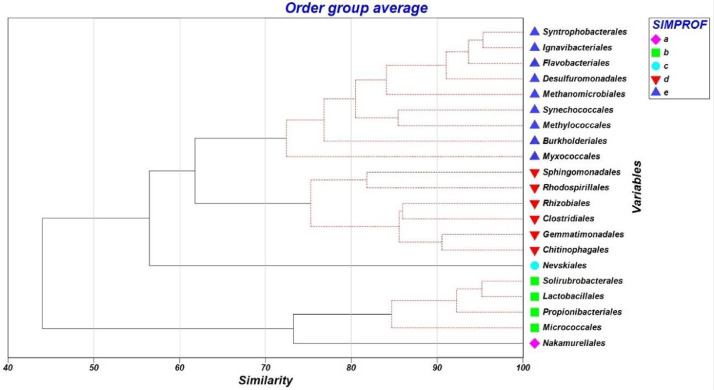
Dendrogram of similarity of bacterial orders in surface sediment of lagoons with fish potential at 70% accumulated contribution, according to SIMPROF analysis.

**Fig. 4:**
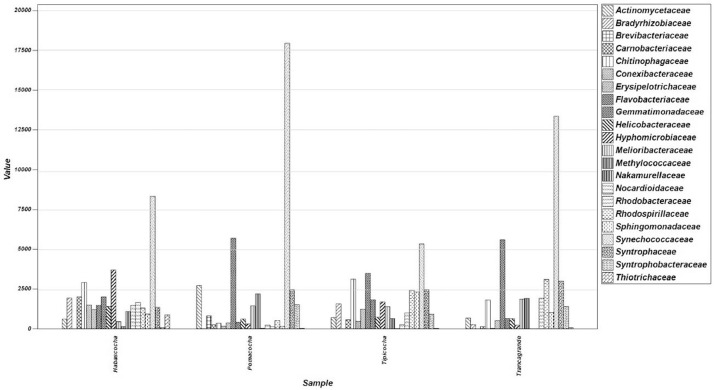
Distribution of bacterial families in surface sediment of ponds with fish potential at 70% contribution.

Table 1 shows the abundance of bacteria in surface sediments of lagoons with fish potential in the Central Andes of Peru, according to phylum, obtained through high performance sequencing. Table 2 shows the mean abundance and percentage contribution of phyla bacteria to the differentiation or similarity between groups, according to the SIMPER analysis. Phylum Actinobacteria presented the highest percentage of contribution to the bacterial communities (29.20%), followed by Cyanobacteria (16.11%) and Proteobacteria (14.66%). The grouping of bacterial orders by SIMPROF analysis, reported five statistically different groups in relation to the number and site of sampling (Fig. 3). The distribution of bacterial families in surface sediments of ponds with fish potential at 70% contribution by SIMPER analysis is shown in Fig. 4.

**Table 1:** Abundance of bacteria in surface sediment of lagoons with fish potential in the Central Andes of Peru, according to phylum.

Phylum	Habascocha	Pomacocha	Tipicocha	Trancagrande
Acidobacteria	2668	2901	3284	1023
Actinobacteria	20,234	13,410	8668	3407
Aquificae	83	1	19	7
Armatimonadetes	23	15	66	18
Bacteroidetes	7001	10,302	10,980	12,591
Caldiserica	20	76	87	74
Candidatus Cloacimonetes	34	22	70	53
Candidatus Korarchaeota	0	0	4	6
Candidatus Saccharibacteria	473	306	578	278
Chlamydiae	15	110	81	61
Chlorobi	14	69	142	51
Chloroflexi	1740	2234	2260	2964
Chrysiogenetes	1	0	0	0
Crenarchaeota	0	0	0	1
Cyanobacteria	13,986	20,855	10,245	22,762
Deferribacteres	0	4	4	0
Deinococcus Thermus	190	335	653	250
Dictyoglomi	112	758	733	1097
Elusimicrobia	2	1	2	2
Euryarchaeota	319	2534	2837	2668
Fibrobacteres	12	32	25	41
Firmicutes	8616	5975	7613	4841
Fusobacteria	35	43	65	55
Gemmatimonadetes	2008	407	1818	654
Ignavibacteriae	442	1440	1418	1876
Kiritimatiellaeota	2	3	2	2
Nitrospirae	653	116	1761	82
Planctomycetes	45	59	55	148
Proteobacteria	58,539	55,169	66,426	64,971
Spirochaetes	108	447	469	612
Synergistetes	16	38	52	74
Tenericutes	147	116	198	134
Thaumarchaeota	7	2	66	3
Thermodesulfobacteria	173	329	353	459
Thermotogae	14	15	13	19
Verrucomicrobia	636	516	969	814

**Table 2:** Mean abundance and percentage contribution of bacterial phyla in lagoon sediment with fish potential in the Central Andes of Peru, according to SIMPER analysis.

Phylum	Av. dissim	Contrib.%	Cumulative%	Mean A	Mean B
Actinobacteria	4.90	29.20	29.20	20,200	8500
Cyanobacteria	2.70	16.11	45.30	14,000	18,000
Proteobacteria	2.46	14.66	59.97	58,500	62,200
Bacteroidetes	1.79	10.68	70.65	7000	11,300
Firmicutes	1.03	6.17	76.81	8620	6140
Euryarchaeota	0.99	5.88	82.69	319	2680
Ignavibacteriae	0.47	2.83	85.52	442	1580
Gemmatimonadetes	0.44	2.62	88.14	2010	960
Acidobacteria	0.35	2.06	90.21	2670	2400
Dictyoglomi	0.31	1.87	92.07	112	863
Chloroflexi	0.31	1.86	93.93	1740	2490
Nitrospirae	0.31	1.84	95.77	653	653
Spirochaetes	0.17	1.00	96.77	108	509
Deinococcus Thermus	0.09	0.55	97.32	190	413
Verrucomicrobia	0.09	0.52	97.85	636	766
Thermodesulfobacteria	0.09	0.52	98.36	173	380
Candidatus Saccharibacteria	0.07	0.39	98.75	473	387
Aquificae	0.03	0.18	98.93	83	9
Chlorobi	0.03	0.18	99.12	14	87.3
Chlamydiae	0.03	0.17	99.29	15	84
Caldiserica	0.02	0.15	99.44	20	79
Planctomycetes	0.02	0.11	99.54	45	87.3
Synergistetes	0.02	0.10	99.64	16	54.7
Tenericutes	0.01	0.08	99.72	147	149
Thaumarchaeota	0.01	0.06	99.77	7	23.7
Candidatus Cloacimonetes	0.01	0.06	99.83	34	48.3
Fibrobacteres	0.01	0.05	99.88	12	32.7
Fusobacteria	0.01	0.05	99.93	35	54.3
Armatimonadetes	0.01	0.05	99.97	23	33
Candidatus Korarchaeota	0.00	0.01	99.98	0	3.33
Deferribacteres	0.00	0.01	99.99	0	2.67
Thermotogae	0.00	0.01	100.00	14	15.7
Chrysiogenetes	0.00	0.00	100.00	1	0
Kiritimatiellaeota	0.00	0.00	100.00	2	2.33
Elusimicrobia	0.00	0.00	100.00	2	1.67
Crenarchaeota	0.00	0.00	100.00	0	0.333

## 2 Experimental design, materials and methods

### 2.1 Sediment sampling

Surface sediment samples (10 cm) were collected from four inland water fish (*Oncorhynchus mykiss*) culture ponds in November 2019. Sediment samples from each lagoon were conditioned in airtight plastic bags and transported on ice to the Universidad Nacional de Tumbes laboratory for analysis [4].

### 2.2 DNA extraction, 16S rRNA genes PCR amplification and sequencing

DNA extraction was performed from 0.5 g sample using the PrestoTM Soil DNA Extraction Kit, in accordance with the manufacturer's instructions and standard protocols. DNA concentration and quality were determined using a NanodropTM ONe quantification spectrophotometer (Thermo Fisher Scientific, Massachusetts, USA) obtaining ranges from 0.3 to 88.5 ng/µl.

PCR amplification was performed using the Gene One and GE Healthcare Life Sciences kits by mixing 1 µl of the 16S rRNA F universal primer, 1 µl of the 16S rRNA R universal primer, 22 µl of the PCR mix (containing premix buffer, MgCl2, dNTPs and taqPolymerase) and 1 µl DNA sample obtaining a total reaction volume of 25 µl. Primers 27 F (5′-AGAGTTGATCCTGGCTCAG-3′) and 1392R (5′-GGTACCTTGTACGACTT-3′) were used and amplified for a product of about 1365 bp. Bacterial sequencing of the 16S rRNA amplicon was performed using the standard next-generation Illumina MiSeq [5], [6], [7], [8]. The construction of the library was carried out commercially (ADMERA HEALTH LLC, USA).

### 2.3 Bioinformatic analysis of sequence readings

The FASTQ files generated by the program FASTQC v0.11.9 were processed to know the length of the readings, the quality of the bases and the percentage of nucleotide bases. Subsequently, quality filtering and removal of regions of the primer and adapters present in the readings was performed using the Trimmomatic v0.39 program [9] with minimum trimming values of Q30 and trimming of readings below 30 bp. All individual reads were greater than 150,000 per isolate with a read length of 251 nucleotides and a quality value of each sequenced base greater than 30. The taxonomic analysis was performed using the program [10], based on the database minikraken_20,171,019_4GB. This program also handles multiple scripts for circular representation. Finally, operational taxonomic units were identified and abundances calculated [11,12].

### 2.4 Statistical analysis

Similarity percentage analysis (SIMPER) was performed to calculate the relative contribution of each taxon to the overall average dissimilarity observed between two or more groups of taxonomic assemblages. The groups were defined on the basis of a preliminary similarity profile clustering analysis (SIMPROF) of the same taxonomic occurrence data set [13]. The SIMPROF analysis allowed to test the multivariate structure within groups of samples. Square-root transformed abundances were used to calculate Bray Curtis similarities [14], showing patterns between samples determined by significant similarity measurements (*p* < 0.05), using clustering and ordering [15]. These analyses were performed in the Primer V7.

### 2.5 Nucleotide sequence access numbers

The 16S rRNA gene sequences reported in this study were sent to the GenBank database with the access number PRJNA657251 (https://www.ncbi.nlm.nih.gov/sra/PRJNA657251).

## Declaration of Competing Interest

The authors declare that they have no known competing financial interests or personal relationships that could have appeared to influence the work reported in this paper.

## Appendix Supplementary materials

Image, application 1
